# The Effect of Telehealth on Quality of Life and Psychological Outcomes Over a 12-Month Period in a Diabetes Cohort Within the Whole Systems Demonstrator Cluster Randomized Trial

**DOI:** 10.2196/diabetes.7128

**Published:** 2017-09-01

**Authors:** Shashivadan P Hirani, Lorna Rixon, Martin Cartwright, Michelle Beynon, Stanton P Newman, WSD Evaluation Team

**Affiliations:** 1 Centre for Health Services Research School of Health Sciences University of London London United Kingdom

**Keywords:** telehealth, self-monitoring, health-related quality of life: diabetes-specific quality of life

## Abstract

**Background:**

Much is written about the promise of telehealth and there is great enthusiasm about its potential. However, many studies of telehealth do not meet orthodox quality standards and there are few studies examining quality of life in diabetes as an outcome.

**Objective:**

To assess the impact of home-based telehealth (remote monitoring of physiological, symptom and self-care behavior data for long-term conditions) on generic and disease-specific health-related quality of life, anxiety, and depressive symptoms over 12 months in patients with diabetes. Remote monitoring provides the potential to improve quality of life, through the reassurance it provides patients.

**Methods:**

The study focused on participant-reported outcomes of patients with diabetes within the Whole Systems Demonstrator (WSD) Telehealth Questionnaire Study, nested within a pragmatic cluster-randomized trial of telehealth (the WSD Telehealth Trial), held across 3 regions of England. Telehealth was compared with usual-care, with general practice as the unit of randomization. Participant-reported outcome measures (Short-Form 12, EuroQual-5D, Diabetes Health Profile scales, Brief State-Trait Anxiety Inventory, and Centre for Epidemiological Studies Depression Scale) were collected at baseline, short-term (4 months) and long-term (12months) follow-ups. Intention-to-treat analyses testing treatment effectiveness, were conducted using multilevel models controlling for practice clustering and a range of covariates. Analyses assumed participants received their allocated treatment and were conducted for participants who completed the baseline plus at least one follow-up assessment (n=317).

**Results:**

Primary analyses showed differences between telehealth and usual care were small and only reached significance for 1 scale (diabetes health profile-disinhibited eating, *P*=.006). The magnitude of differences between trial arms did not reach the trial-defined minimal clinically important difference of 0.3 standard deviations for most outcomes. Effect sizes (Hedge's g) ranged from 0.015 to 0.143 for Generic quality of life (QoL) measures and 0.018 to 0.394 for disease specific measures.

**Conclusions:**

Second generation home-based telehealth as implemented in the WSD evaluation was not effective in the subsample of people with diabetes. Overall, telehealth did not improve or have a deleterious effect quality of life or psychological outcomes for patients with diabetes over a 12-month period.

## Introduction

The increasing demands of diabetes care on health resources in many countries [1] has led to the development of innovative information-communication-technology–based interventions that facilitate patient self-care and the monitoring and communication of disease status to health care professionals [2]. The range of technologies includes disc- and computer-based systems [3], Web-based interventions [4,5], mobile apps [6], remote monitoring systems [7,8], and combinations of these. One system gaining traction in the last 10 years is telehealth, which involves the remote exchange of physiological or symptom data between a patient and health care professional [9,10]. Algorithms within systems logging the data sent can alert health care professionals when disease-specific clinical parameters are breached; allowing the potential for earlier intervention, which can reduce the frequency with which expensive hospital-based care is required, and thereby improving patient outcomes (eg, reducing avoidable hospitalizations, improving clinical parameters) and health-related quality of life (HRQoL), the latter of which is the focus of this paper.

Primary studies and systematic reviews that have examined the effect of telehealth on HRQoL in people with diabetes, typically conclude that telehealth leads to QoL improvements, potentially because of improved care processes and health status, and reductions in worry about timely interventions as physiological and physical status are being monitored by health care professionals. For example, one potential pathway by which telehealth impacts patient outcomes is the increased feelings of reassurance participants get by being more closely monitored by the health care team, the other potential mediating mechanism it that increasing knowledge of the condition and increasing confidence leads to improvements in self-care behaviors, such as checking feet regularly, and so on [11]. However, in 1 systematic review [12], it was not possible to quantitatively synthesize the evidence on patient outcomes due to the heterogeneity of the patient-reported outcomes (PROMS). The authors found 5 studies that measured HRQoL, and of these 4 reported no significant differences [13-16], which is consistent with a recent randomized controlled trial (RCT) that found no differences in PROMs in a UK-based telehealth service [7]. In contrast, Chumbler et al. [17] found statistically significant improvements in 3 of 8 short-form (SF-36) subscales (role functioning, bodily pain, and social functioning) after 1 year of home telehealth. A further source of heterogeneity in the studies is the mixture of generic versus disease specific measures of HRQoL.

Few studies, however, have examined psychological distress. This is despite some contention about whether telehealth, despite monitoring benefits, can have potentially detrimental effects increasing patient burden and distress [18], through greater isolation and reduced face-to-face contact with health care professionals [19], and at times low acceptability of telehealth [20].

The evidence base for telehealth in people with diabetes is characterized by methodologically weak studies that generate equivocal findings and the studies have been critiqued for their variability in quality (small samples, poor methodology, few RCTs) and heterogeneity (in systems examined and outcomes measured) that has made the information produced difficult to interpret or synthesize [12]. The effectiveness of telehealth, in terms of QoL benefits, has yet to be substantiated in high-quality trials. Furthermore, few studies have used diabetes-specific QoL instruments, which are more sensitive to changes in this population than generic QoL measures, and few studies have extended the psychosocial outcomes to examine anxiety and depression.

The current study was part of the Whole Systems Demonstrator (WSD) programme, commissioned by the UK Department of Health. A previous paper has already reported on the effect of telehealth on HbA1c control in the larger diabetes trial cohort [21]. This paper reports on a subsample of the cohort providing data on the PROMs. It aimed to address the inconsistencies in data observed in previous research in telehealth and patient-reported QoL outcomes, and evaluated the effectiveness of telehealth in a sample of people with diabetes, examining its effect on HRQoL and psychological distress in the short and long term. It was hypothesized that should telehealth demonstrate significant improvements in QoL measures, these would be detected in disease-specific measures to a greater degree than generic QoL measures; and that telehealth would significantly improve psychological distress due to the reassurance the monitoring systems would provide to patients.

## Methods

### Design and Randomization

The WSD evaluation was one of the largest trials evaluating telehealth and telecare in the United Kingdom. The detailed protocol and design for the WSD evaluation has been reported elsewhere [22]. Within the evaluation, the WSD Telehealth Trial (n=3230) was a multicenter, pragmatic, cluster-RCT of telehealth across 3 regions in England (Cornwall, Kent, and the London Borough of Newham) with a nested questionnaire study, the WSD Telehealth Questionnaire Study (1573/3230, 48.7%).

Participants in the trial were allocated to a trial arm (ie, telehealth or usual care) using cluster randomization, based on participants’ registration with a general practice. Allocation was balanced for region (WSD site), practice size, deprivation index, non-white proportion and prevalence of diabetes, chronic obstructive pulmonary disease, and congestive heart failure, using an algorithm by the trial statistician. For individual participants, trial arm allocation was maintained from the main trial, through to the questionnaire study and diabetes participant analyses. The WSD Telehealth Questionnaire Study involved a total of 204 general practices recruited across the 3 WSD Sites, of which 111 contributed participants to the diabetes questionnaire analysis; 46.8% (52/111) in the control and 53.2% (59/111) in the intervention trial arm.

Participants diagnosed with diabetes were recruited between May 2008 and December 2009 from 4 primary care trusts across the 3 WSD regions. Final 12-month follow-ups were conducted in December 2010. Participants in the trial were invited to take part in a nested questionnaire study measuring PROM. Neither participants nor assessors could be blinded to trial arm allocation, due to the nature of the intervention. Participants not allocated to receive telehealth were informed that they would be offered the technology at the end of the trial period, following a reassessment of need.

The study protocol was approved by the Liverpool Research Ethics Committee (Reference number: 08/H1005/4). Full consent procedures are available in the protocol papers by Bower et al. [22] and Cartwright et al. [8]. In brief, practices at each of the sites signed memorandums of agreement to participate in the trial. Telehealth trial participants provided signed, informed consent to share data with the trial team; with those going onto the questionnaire study, providing further signed consent.

### Participants

Adult patients at participating general practices were deemed eligible for the study if they were diagnosed with diabetes according to: (1) the Quality Outcomes Framework register in primary care, (2) a confirmed diagnosis in medical records as indicated by general practice Read Codes or the International Statistical Classification of Diseases and Related Health Problems-10 codes, or (3) confirmation of diabetes by a clinician involved in their care. Participants were not excluded because of additional co-morbidities. However, they were required to have sufficient cognitive capacity and English language skills to complete a self-reported questionnaire and use telehealth kit.

Participants were also required to have a landline telephone for broadband Internet connection, and in the London Borough of Newham an additional requirement was a television set. Local WSD project teams paid for financial costs associated with the telehealth (including phone calls to the monitoring centers, broadband service, and data transmission to the monitoring centers).

### Telehealth Treatment: Intervention Arm

WSD sites delivered variations of a ‘second generation’ telehealth [23] that had a focus on monitoring vital signs, symptoms, and self-management behaviors, and providing health education in common. A full description of the intervention is published elsewhere [8].

In general, participants with diabetes in the trial arm received a glucometer and blood pressure monitor, plus additional peripherals depending on clinical need (eg, weighing scales, pulse oximeter, peak-flow meter, thermometer). The peripheral devices were attached to a home monitoring system comprising a base unit with a liquid-crystal display screen to allow questions about health and educational messages to be transmitted to participants or set-top box that connected to a television allowing symptom questions, educational videos, and a graphic history of clinical readings to be accessed via a dedicated channel. Participants were asked to take measurements via the peripherals on a schedule determined via individual circumstances (eg, daily readings, twice weekly readings).

Data transmitted by participants to a monitoring center were processed via an algorithm for unusual patterns, out of range values, and/or missing data. Contravening a rule triggered an alert to an operator at a monitoring center who would follow a decision tree to determine an appropriate response. The range of responses included: doing nothing—wait and see approach; requesting a repeat reading through the telehealth kit, contacting the participant or their named informal carer, arranging a visit to the participant’s home by their community matron, or referring to another health care service, as appropriate. The intervention arm participants received the telehealth in addition to usual health and social care. At the end of the 12-month trial participants were given the option of keeping telehealth or having it removed from their home.

### Usual-Care Treatment: Control Arm

Participants randomized to the control arm received usual health and social care in line with local protocols for the 12-month duration of the trial (eg, combination of community matrons, district nurses, specialist nurses, general practice, and hospital services based on clinical need). At the end of the trial control participants were offered the installation of telehealth services in their homes, if they were still eligible following a needs assessment.

### Trial Assessment Procedures

Outcome measures were self-completed by participants. At baseline, a trained researcher was on hand to explain or clarify the meaning of particular questions or assist with the completion of the questionnaire. Two further assessments were conducted at short-term follow-up conducted at approximately 4 months (median duration = 128 days; interquartile range [IQR] = 47 days) and a long-term assessment, conducted at approximately 12-months (median duration = 366 days; IQR = 54 days).

The short-term follow-up questionnaire was primarily administered as a postal survey with 1 reminder letter for nonresponders; some participants also received telephone reminders. Long-term follow-up surveys were posted to participants, with nonresponders contacted to arrange home interviews with a trained researcher in line with the baseline protocol. Participants who withdrew from the trial, including intervention participants who asked for the telehealth equipment to be removed before the end of the trial period, were not sent further questionnaires.

### Outcome Measures

Generic and disease-specific HRQoL was assessed by: (1) the SF-12 [24] subscales for physical component summary (PCS), and mental component summary (MCS), (2) EuroQual (EQ-5D) York-Tariff [25], 1990, which produces a summary index over 5 domains (mobility, self-care, usual activities, pain/discomfort, and anxiety/depression), (3) the diabetes health profile (DHP) [26] with subscales measuring psychological distress, barriers to activity and disinhibited eating, and (4) study-specific diabetes HRQoL measures of social marginalization and social conspicuousness. Measures were also taken of anxiety with the brief state trait anxiety inventory (STAI) [27] and depressive symptoms by the 10-item Center for Epidemiologic Studies Depression scale (CESD-10) [28]. Higher scores on the QoL instruments pertained to better QoL and higher scores on the anxiety and depression instruments indicated greater psychological distress.

Demographic information recorded included age, sex, ethnicity, number of co-morbid conditions, and level of education. Participants’ levels of deprivation were allocated using an Index of Multiple Deprivation score [29] as assessed through postcodes.

### Sample Size Calculation

For the disease-specific aspects of the questionnaire study, a power calculation was conducted on the basis of detecting a small effect size, equivalent to a Cohen *d* of 0.3 [30], allowing for an intracluster correlation coefficient of 0.05, power of 80% and *P*<.05. This indicated that between 420 and 520 participants would be required to allow sufficient power to detect this small difference taking account of the cluster design. These numbers were inflated by 10% to allow for the maximum possible increase in sample size due to variable cluster size. The required minimum sample size increased to 550.

### Statistical Methods

*Missing data* rates (at the scale/item level used in analyses) among those returning questionnaires at short and long term were low (≤3%) and were imputed (m=10) using the SPSS MCMC function within each administration. Thereafter, standard multiple imputation procedures were employed [31]. Details of multiple-imputation processes are available from the authors.

#### Sample Characteristics

Frequencies and mean scores are reported for each trial arm at each follow-up. Analyses were conducted on a modified intention-to-treat basis (ie, available case analyses—where data was available for baseline plus at least 1 follow-up point).

#### Detecting Telehealth Effects

Repeated measures in each outcome over the 1-year follow-up period were analyzed with linear mixed-effects modeling procedures to detect: trial-arm effects, time effects, and their interaction. This method took account of the hierarchy within the data observations (ie, assessment points, were nested within participants, nested within general practices). Data are presented as estimated marginal means (EMMs) with standard errors (SE).

Covariates to adjust for case-mix differences between trial arms were: age, sex, deprivation, ethnicity, co-morbidities, highest education level, WSD site, number of devices, and baseline outcome score. For all parameter tests the alpha level was set to .05; Sidak’s adjustment was used to compensate for post hoc multiple comparisons; 95% confidence intervals (CI) were used to account for the uncertainty in the estimates. Effect sizes for the trial arm effects of each outcome were reported as Hedge’s g. Analyses were conducted in SPSS v19.

## Results

### Sample Recruitment and Attrition

**Figure 1:**
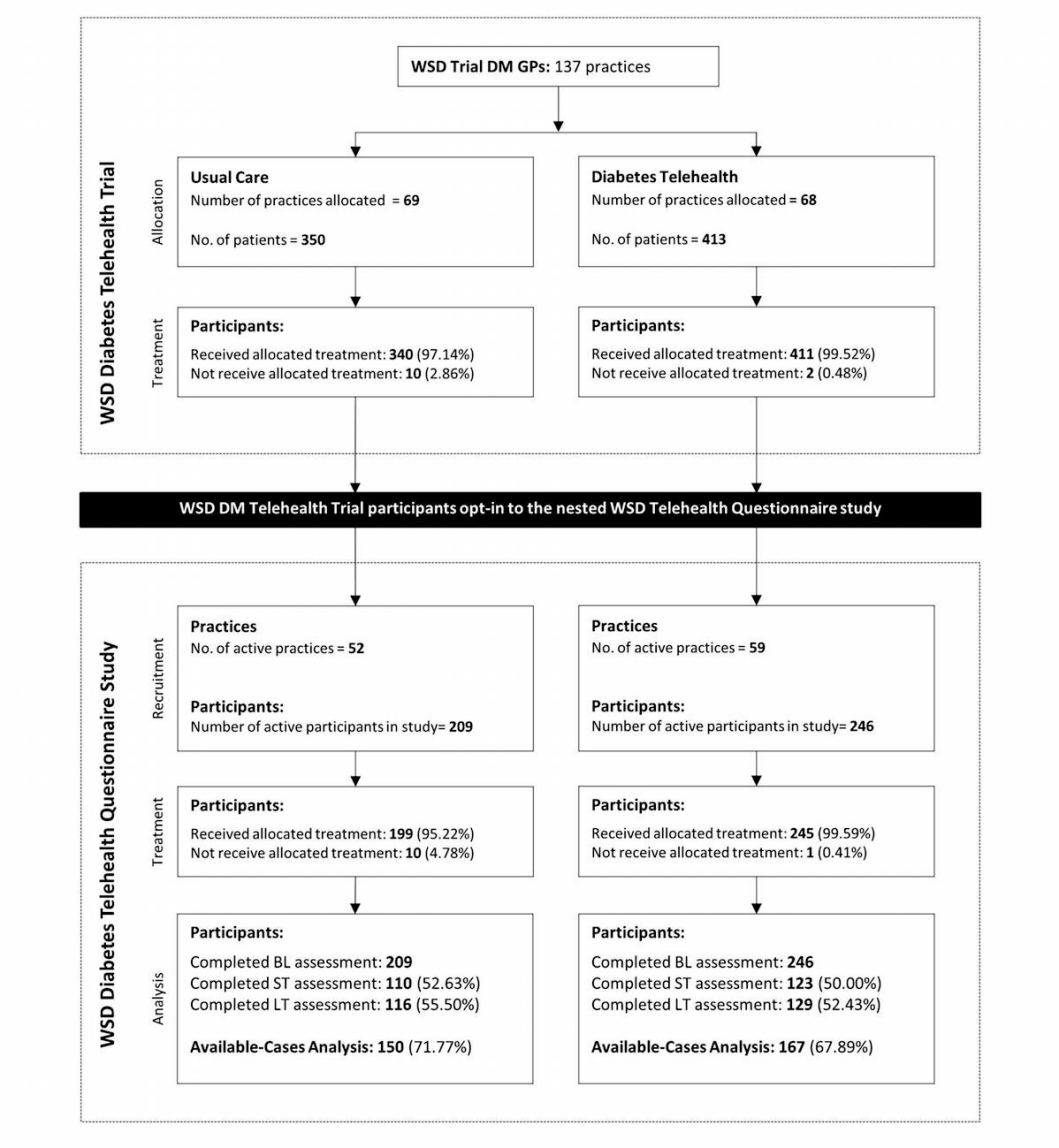
All sites CONSORT diagram for the WSD Telehealth Diabetes Trial.

Of the 3230 participants in the WSD Telehealth Trial, 23.6% (763/3230) were indexed as participants with diabetes. Of the 1573 participants in the nested telehealth questionnaire study, 28.9% (455/1573) were people with diabetes; of these 54.1% (246/455) were in the intervention arm and 45.9% (209/455) were in the usual care arm. Figure 1 shows participants per trial arm within the questionnaire study.

### Sample Characteristics

Baseline sample characteristics by trial arm of the 455 questionnaire participants are reported in Table 1. The mean age of the sample was approximately 65 years with most participants being of white, British/Irish ethnicity. Most participants came from the London Borough of Newham WSD Site, and were mainly male. The sample had on average 2 co-morbid conditions and the majority (247/455, 54.3%) had received little formal education. On average, the intervention group received just short of 3 telehealth devices. In the telehealth arm 237 glucometers were distributed, with 232 blood pressure monitors, 185 weight scales, and 56 pulse oximeters.

Unadjusted means by trial arm for baseline PROM data are presented in Table 1. CIs calculated around each mean suggested differences between the telehealth and usual care groups were not statistically significant in any measure at baseline.

Physical and mental health component scores for the SF12 and EQ5D health status measures were lower/equal than population averages, but were considered appropriate for a population in this age range with long-term conditions [24,25]. Both anxiety and depression levels were slightly high with the depression level means close to the cut-off point for screening clinical levels of depression. The diabetes health profile (DHP) scales and additional social-based HRQoL scales (social conspicuousness and social marginalization) did not indicate problems with diabetes specific QoL, and showed a relatively well-functioning long-term condition sample.

**Table 1:** Site, sex, and ethnicity frequencies per trial arm of the questionnaire participants with diabetes.

			Intervention (n=246) n (%)		Control (n=209) n (%)		Total (N=455) n (%)
**Site^a^**						
	Cornwall		64 (26.0)		55 (26.3)		119 (26.2)
	Kent		44 (17.9)		46 (22.0)		90 (19.8)
	London Borough of Newham		138 (56.1)		108 (51.7)		246 (54.1)
**Sex^a^**						
	Female		115 (46.7)		84 (40.2)		199 (43.7)
	Male		131 (53.3)		125 (59.8)		256 (56.3)
**Ethnicity**						
	Non-white		79 (32.1)		72 (34.4)		151 (33.2)
	White British / Irish		167 (67.9)		137 (65.6)		304 (66.8)

^a^Not multiply imputed.

**Table 2:** Baseline sample characteristics per trial arm of questionnaire participants with diabetes.

		Intervention (n=246) Mean (standard error)		Control (n=209) Mean (standard error)		Total (N=455) Mean (standard error)
Age, years^a^	64.72 (.874)		65.27 (.875)		64.97 (.620)	
Deprivation score	35.12 (.957)		33.70 (.896)		34.47 (.661)	
Number of Comorbidities^a^	2.11 (.118)		2.17 (.128)		2.14 (.087)	
Amount of telehealth - number of devices^a^	2.89 (.047)		0.16 (.051)		1.64 (.073)	
Level of education	0.83 (.078)		0.97 (.088)		0.89 (.059)	
SF-12^b^ Physical Component Scale	30.31 (0.61)		30.75 (0.66)		30.51 (0.45)	
SF-12 Mental Component Scale	35.27 (0.57)		35.38 (0.61)		35.32 (0.42)	
EQ5D^c^ scale	0.50 (0.02)		0.52 (0.03)		0.51 (0.02)	
State Anxiety scale (Brief STAI^d^)	11.37 (0.29)		10.92 (0.31)		11.16 (0.21)	
Depression scale (CESD10^e^)	11.10 (0.44)		10.32 (0.45)		10.74 (0.32)	
Disinhibited Eating DHP^f^-subscale	42.44 (1.28)		41.39 (1.24)		41.96 (0.90)	
Psychological Distress DHP-subscale	23.84 (1.54)		24.03 (1.66)		23.93 (1.12)	
Barriers to Activity DHP-subscale	32.58 (1.44)		32.81 (1.65)		32.69 (1.08)	
Social Impact DHP-subscale	12.20 (1.03)		11.79 (1.04)		12.01 (0.73)	
Social Marginalization DHP-subscale	13.61 (1.09)		13.64 (1.13)		13.62 (0.79)	
Social Conspicuousness DHP-subscale	10.30 (1.21)		9.22 (1.17)		9.81 (0.84)	

^a^Not multiply imputed.

^b^Short-Form 12 item survey.

^c^EuroQual EQ-5D.

^d^State Trait Anxiety Inventory.

^e^Center for Epidemiologic Studies Depression scale.

^f^Diabetes Health Profile.

**Table 3:** Parameter estimates for trial arm and time in the linear mixed-effects modeling analysis for available cases (n=317).

	Trial Arm			Time				Time × Trial Arm			
	Estimate	Standard error	Significance		Estimate	Standard error	Significance		Estimate	Standard error	Significance
SF 12 - PCS^a^	0.338	1.801	0.851		0.335	0.703	0.634		−0.298	0.976	0.760
SF 12 - MCS^b^	1.806	1.776	0.309		−0.024	0.639	0.970		0.024	0.881	0.978
EQ5D^c^	0.087	0.068	0.201		0.021	0.026	0.417		−0.050	0.036	0.167
Anxiety	−0.232	1.053	0.825		0.604	0.415	0.146		−0.250	0.568	0.660
Depression	0.488	1.364	0.720		0.100	0.528	0.849		−0.189	0.734	0.797
Psychological Distress	−1.161	4.63	0.802		0.042	1.826	0.982		3.491	2.64	0.187
Barriers to Activity	3.561	4.524	0.431		1.779	1.694	0.294		−1.293	2.441	0.596
Disinhibited Eating	10.674^d^	3.847^d^	0.006^d^		1.754	1.649	0.287		−0.649	2.386	0.786
Social Marginalization	−3.476	3.677	0.345		−0.703	1.493	0.638		2.288	2.087	0.273
Social Conspicuousness	−2.275	3.374	0.500		0.764	1.427	0.592		1.610	1.973	0.415

^a^Short Form 12-item Physical Component Summary.

^b^Short Form 12-item Mental Component Summary.

^c^EuroQual EQ-5D.

^d^Significant effects (*P*<.05).

### Detecting Telehealth Effects

**Figure 2:**
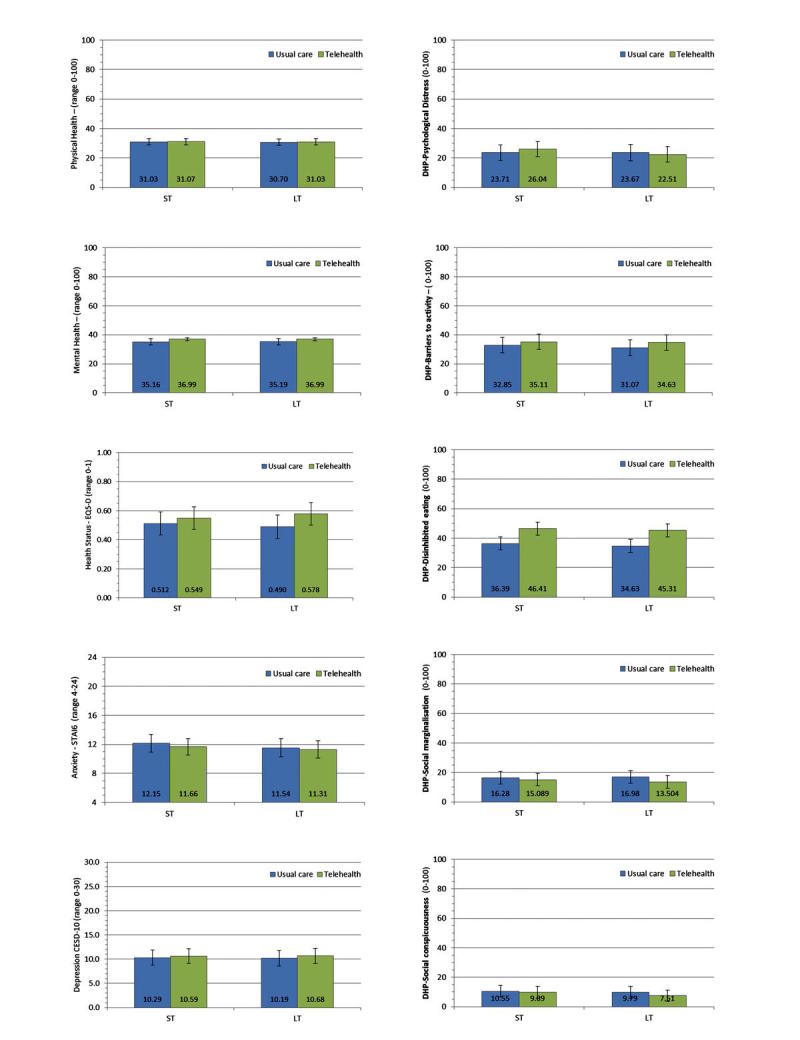
Covariate adjusted mean scores (with 95% confidence interval) for each patient-reported outcomes by trial arm. ST: short-term, LT: long-term.

Table 3 presents key parameter estimates for the effect of trial arm, time, and their interaction from linear mixed-effects modeling analyses (adjusting for case-mix) conducted for each outcome (parameters for covariates are not presented). Only 1 effect from the 10 PROMs was significant, on the DHP disinhibited eating subscale—where a significant trial arm effect was detected. Adjusted means (EMMs) for each outcome measure by trial arm and time point are presented in Figure 2, (for unadjusted means see Multimedia Appendix 1).

**Figure 3:**
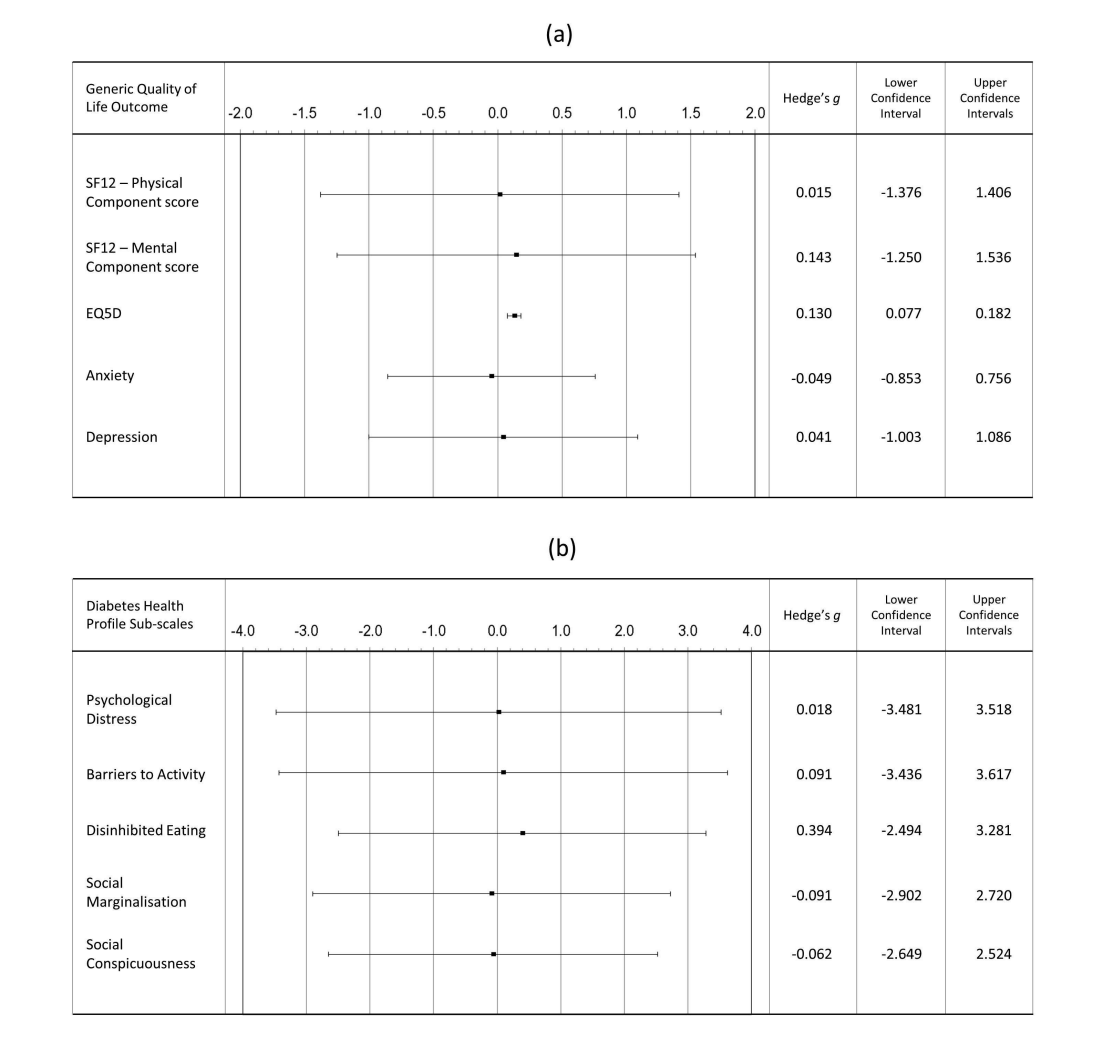
Effect size estimates for the (a) generic quality of life (QoL) and psychosocial well-being outcomes, and (b) the disease-specific QoL outcome measures.

Parameter estimates indicate that being a member of the telehealth intervention trial arm provides an approximately 10-point advantage on the DHP disinhibited eating scale (after the intracluster correlation, all covariates and data hierarchy are taken into account), as indicated by EMM of the DHP disinhibited eating scale of the control (mean=35.512, SE=2.074) and intervention arms (mean=45.861, SE=2.086; *F*_1,757.625_=7.697, *P*=.006). Effect-size estimates reveal this to be a small to medium effect, however the effect size had large 95% CIs, which crossed the 0 border (Figure 3).

The only measure to have ES CI that did not cross the 0 mark was the EQ-5D. However, the estimated effect size was very small (Cohen criteria) and the upper CI did not exceed 0.2, suggesting that although this is a robust ES, its magnitude is unlikely to have a substantial clinical impact.

Sensitivity analyses (ie, analyses per protocol, with complete cases, and/or excluding covariates) indicated similar trends in effects.

## Discussion

### Principal Findings

This analysis examined the effect of telehealth on participant reported outcomes in a relatively large sample of patients with diabetes, who partook in the WSD telehealth trial. Overall scores for the sample indicate that physical and mental health component scores for the SF12 and EQ5D health status measures were similar to a population with diabetes. Both anxiety and depression levels were slightly high, with the depression level means close to the cut-off point for screening clinical levels of depression. The DHP scales and additional social-based HRQoL scales (social conspicuousness and social marginalization) did not indicate problems with diabetes-specific QoL, and indicated a relatively well-functioning long-term condition diabetes sample.

The telehealth group means generally indicated marginally better generic HRQoL outcomes for the telehealth group; and the usual care better marginally better outcomes on the disease-specific and psychological distress scales. However, overall these differences did not reach statistical significance, with the results suggesting that telehealth, relative to usual care, does not significantly impact upon patients HRQoL (generic and disease-specific) or their psychological distress over a period of 12 months. Nor does the status of these participants’ PROMs greatly alter over the 12-month period, regardless of their treatment group.

The only significant effect across the analyses of the PROMs was found on the DHP disinhibited eating subscale—where a significant trial arm effect was detected. Parameter estimates indicated that being a member of the telehealth intervention trial arm provided an approximately 10-point increase on the DHP disinhibited eating scores. This may have indicated that with telehealth patients are more likely to undertake disinhibited eating (eg, lack eating control, emotional eating), perhaps as a response to knowing that should any effects of lacking eating control become extreme, they are being monitored and health care professionals will be able to suitably intervene. The provision of telehealth has the potential to increase individual’s empowerment and self-care behaviors to manage their conditions through remote monitoring, rather than leading to a reliance of health care professional control. The mechanisms of such unexpected negative effects need further investigation in relation to theoretical constructs of behavioral change. Furthermore, effect-size estimates revealed this effect on disinhibited eating to be a small to medium effect, with large CIs that crossed the 0 border, indicating poor reliability in this estimate.

The only outcome with an effect size CI that appeared robust was with the EQ5D measure. However, the magnitude of this effect indicated that it would unlikely be clinically significant. The lack of effects on these PROM could also be because patients with diabetes are used to monitoring their conditions, in terms of checking blood glucose, monitoring their diets, and activity levels [32,33], and the potential benefits of the additional remote connections to health care professionals do not add value to their self-monitoring behaviors.

Despite lack of effects on PROMs, the WSD diabetes cohort showed modest gains in glycemic control [21], which was similar to another UK-based RCT [7]. There was also evidence that the telehealth trial was effective at reducing hospital admissions and mortality [34]. There were no differences on diabetes specific QoL, self-care behaviors, self-efficacy, which is consistent with recent pragmatic multicenter RCT in the UK [7], and other long-term conditions in the WSD trial [8,35]. However, these results demonstrated no substantial decreases in these outcomes either. To gain improvement in PROMS, the telehealth system may need to be broader than self-monitoring of blood glucose and designed to target the behavioral antecedents to these PROMs in individuals with impaired mood and HRQoL. Telehealth services may need to be more tailored to the individual, so that there is a match between the person and the technology to increase its impact.

This study also examined the use of novel social functioning with diabetes scales of social marginalization and social conspicuousness. Overall, the results showed that there are only small impacts in these 2 areas of social life and that they are not impacted upon by telehealth as delivered in this study. However, it may be the case that non–home-based remote monitoring, other technology-enabled care systems or mobile monitoring [3-6] would have a greater impact in these areas.

### Strengths and Limitations

This clustered RCT addresses many of the methodological limitations identified in previous studies and adds evidence to an important gap in the literature. However, caution is required as although this was a relatively large sample of patients with diabetes compared with past studies, in the available cases analyses, the sample size did fall short of the recommended number required to detect a small effect. Despite recruiting 455 patients at baseline, the required number was not met due to attribution. This highlighted the difficulties in recruiting and maintaining participants in a trial of this size and complexity; nevertheless, a larger sample may help narrow the CIs of effect sizes and identify further statistically significant effects.

Also, the WSD trial was a pragmatic trial, but with associated limitations. While it has good ecologic validity, 1 potential criticism is the number of confounding factors (eg, the nature of the telehealth intervention delivered at each of the regional WSD sites/participating). Like other studies in this area, there is a high risk of selection bias given that the numbers of eligible patients the study sample were drawn from is unknown. Nevertheless, the WSD trial recruited a large number of patients with diabetes, is 1 of very few UK-based studies conducted in the National Health Service, and benefits from high generalizability across different centers, given the inclusion of a many general practices (n=204) delivering telehealth or standard care to patients with diabetes. However, in his study we did not examine differences between patients using insulin as well as oral medication and those who were only using oral medication. It is likely that insulin use will have a greater effect on HRQoL than medications alone, and thus insulin users may have a greater potential for the support via telehealth. This potential impact requires further investigation, especially in relation to the timeframes within which telehealth may have positive impacts upon HRQoL and psychological distress in each group of patients with diabetes.

Importantly, as an RCT, this study did not aim to specifically examine the mechanisms by which telehealth may impact PROMs. The differences in the types of telehealth and how they may differentially affect outcomes needs better investigation—as they likely use different mechanisms for action on HRQoL and psychological distress, making it problematic to compare the effectiveness of trials. Telehealth solutions also need to be described in sufficient detail, to determine how their use in the complex health care environment of diabetes management, may lead to improved HRQoL outcomes. Monitoring and interpreting readings in diabetes self-management is only 1 domain of a complex set of behaviors patients are advised to follow. Thus, the complexity of interventions, including the integrated role of telehealth across services, need to be adequately described with the mediating and moderating variables also examined. Furthermore, additional types of technology that patients with diabetes may use in addition to the telehealth services provided by the general practitioner/local authority also need to be considered, as they may mask effects specific to these services.

### Implications and Future Research

The findings have implications for mainstreaming telehealth. Providing telehealth alone, in the absence of monitoring and enhancing the mediating mechanisms (eg, self-care behaviors, self-efficacy [Cartwright et al. Unpublished data], acceptability [20], and reducing dropout [36]) will not necessarily lead to improvements in HRQoL. In the future, further improvements to these complex interventions maybe required for telehealth to be used as a tool to improve patients’ self-care and HRQoL. For example, evidence-based self-management interventions could be delivered via telehealth to facilitate the management of long-term conditions, such as diabetes and the capability of mobile monitoring may need to be integrated into home-based telehealth packages.

### Conclusions

This study found no substantial impacts of telehealth on either generic or disease-specific HRQoL measures in a population with diabetes. However, this study also demonstrated that there were no substantial decreases in HRQoL with the introduction of telehealth. Coupled with moderate improvements in glycemic control, there is potential promise for telehealth interventions, but more effective telehealth interventions aimed specifically at improving outcomes measured by PROMs are needed. Self-monitoring using telehealth is insufficient to improve PROMS by itself, but we recommend using evidenced based self-management techniques targeting self-care and QoL delivered via telehealth, as a tool to facilitate the delivery of the intervention.

## Appendix

Multimedia Appendix 1 Unadjusted mean scores (with 95% confidence interval) for each patient-reported outcome by trial arm. BL: base line; ST: short term; LT: long term.

## Abbreviations

CESD-10: Center for Epidemiologic Studies Depression scale
CI: confidence interval
DHP: diabetes health profile
EMM: estimated marginal means
EQ5D: EuroQual 5D
HRQoL: health-related quality of life
IQR: interquartile range
MCS: mental component summary
PCS: physical component summary
PROMS: patient-reported outcomes
RCT: randomized controlled trial
SE: standard errors
SF: short-form
STAI: state trial anxiety inventory
WSD: whole systems demonstrator
