# Supplementary figures and images for: The Effect of Telehealth on Quality of Life and Psychological Outcomes Over a 12-Month Period in a Diabetes Cohort Within the Whole Systems Demonstrator Cluster Randomized Trial
Source: JMIR Diabetes. 2017 Sep 1;2(2):e18. doi: 10.2196/diabetes.7128 (PMC6238866; doi:10.2196/diabetes.7128)

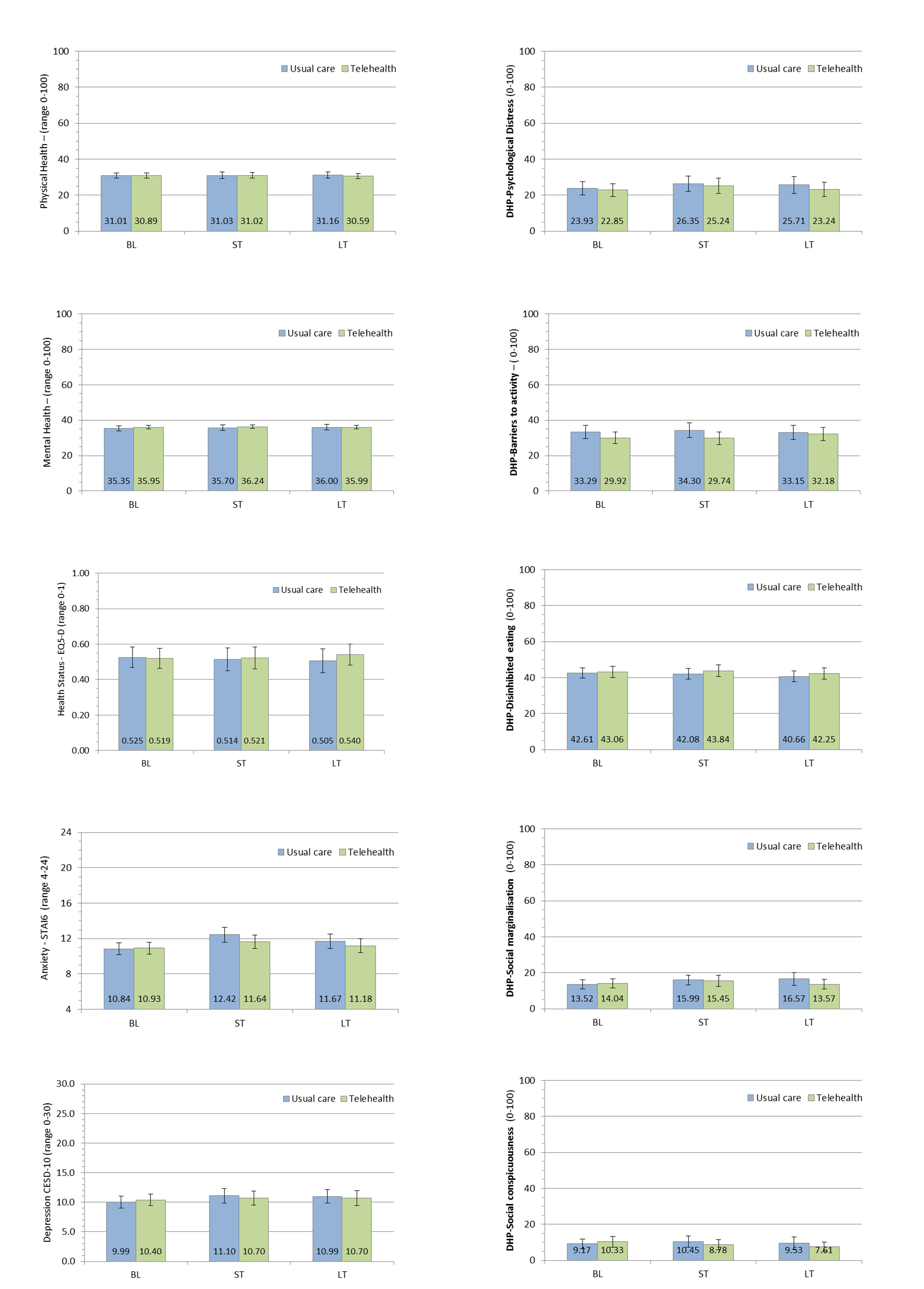

Supplement: Multimedia Appendix 1 [file diabetes_v2i2e18_app1.jpg]
